# A melanoma subtype with intrinsic resistance to BRAF inhibition identified by receptor tyrosine kinases gene-driven classification

**DOI:** 10.18632/oncotarget.3007

**Published:** 2015-01-30

**Authors:** Matteo Dugo, Gabriella Nicolini, Gabrina Tragni, Ilaria Bersani, Antonella Tomassetti, Valentina Colonna, Michele Del Vecchio, Filippo De Braud, Silvana Canevari, Andrea Anichini, Marialuisa Sensi

**Affiliations:** ^1^ Functional Genomics and Bioinformatics, Department of Experimental Oncology and Molecular Medicine, Fondazione IRCCS Istituto Nazionale dei Tumori, Milan, Italy; ^2^ Unit of Immunobiology of Human Tumors, Department of Experimental Oncology and Molecular Medicine, Fondazione IRCCS Istituto Nazionale dei Tumori, Milan, Italy; ^3^ Department of Pathology, Fondazione IRCCS Istituto Nazionale dei Tumori, Milan, Italy; ^4^ Unit of Molecular Therapies, Department of Experimental Oncology and Molecular Medicine, Fondazione IRCCS Istituto Nazionale dei Tumori, Milan, Italy; ^5^ Department of Clinical Oncology, Fondazione IRCCS Istituto Nazionale dei Tumori, Milan, Italy

**Keywords:** melanoma subtypes, receptor tyrosine kinases, BRAF, BRAF inhibitors, drug resistance

## Abstract

Dysregulation of receptor tyrosine kinases (RTKs) contributes to several aspects of oncogenesis including drug resistance. In melanoma, distinct RTKs have been involved in BRAF inhibitors (BRAFi) resistance, yet the utility of RTKs expression pattern to identify intrinsically resistant tumors has not been assessed. Transcriptional profiling of RTKs and integration with a previous classification, reveals three robust subtypes in two independent datasets of melanoma cell lines and one cohort of melanoma samples. This classification was validated by Western blot in a panel of patient-derived melanoma cell lines. One of the subtypes identified here for the first time displayed the highest and lowest expression of *EGFR* and *ERBB3*, respectively, and included *BRAF*-mutant tumors all intrinsically resistant to BRAFi PLX4720, as assessed by analysis of the Cancer Cell Line Encyclopedia pharmacogenomic study and by *in vitro* growth inhibition assays. High levels of EGFR were detected, even before therapy, in tumor cells of one of three melanoma patients unresponsive to BRAFi. Use of different pharmacological inhibitors highlighted the relevance of PI3K/mTOR signaling for growth of this PLX4720-resistant subtype. Our results identify a specific molecular profile of melanomas intrinsically resistant to BRAFi and suggest the PI3K/mTOR pathway as a potential therapeutic target for these tumors.

## INTRODUCTION

The discovery of the *BRAF^V600E^* substitution as the most common genetic event in melanoma [1] rapidly led to the clinical development of selective ATP-competitive RAF kinase inhibitors (i.e. Vemurafenib, Dabrafenib) targeting the mutant BRAF protein [2, 3]. These two drugs gained FDA approval, based on evidence for significant improvement in response rates and in progression free survival, compared to chemotherapy, in randomized phase III trials [4, 5]. Despite these remarkable clinical results, acquired resistance invariably develops in most patients, including those showing an initial strong regression of tumor burden [4, 6]. Furthermore, approximately 1 in 5 patients with BRAF mutant melanoma shows progression at first assessment during treatment, due to intrinsic/primary resistance in their tumors [6, 7] indicating that the mutational status of the target oncogene is insufficient to predict responsiveness to therapy. The identification of molecular features associated with primary resistance to mutant BRAF targeting will enable identification of melanoma patients likely to fail treatment. To this end, gene expression profiling provides powerful means of classifying tumors based on their underlying biology [8–11]. In melanoma, two divergent major subtypes, consistently identified by several authors [12–16], could be classified according to the “Melanoma Phenotype-Specific Expression” (MPSE) signature [17]. This signature includes the melanocyte master regulator microphthalmia-associated transcription factor (*MITF*) and many of its known targets [18]. Low expression of *MITF* and MITF-regulated genes and high expression of genes involved in motility and invasiveness, including *AXL*, defines the subtype named invasive, while the proliferative one is defined by opposite expression of these genes [17]. Indeed, we have previously shown that AXL is highly expressed, at the protein level, in human melanoma cell lines and clinical samples which lacks expression of MITF and of MITF-targets [19]. The antagonistic *MITF/AXL* transcriptional profile was recently linked to intrinsic resistance to RAF and MAPK pathway inhibitors [20]. Thus, higher levels of *MITF* and correlated genes were found in BRAF mutant tumors sensitive to the BRAF inhibitor (BRAFi) PLX4720 and to the MEK inhibitor (MEKi) AZD6244, whereas resistant lines were associated to high NF-κB activity and expression of *AXL* and correlated genes [20].

Subtype-specific expression of key signaling proteins like AXL and other RTKs is also central to the signaling pathways inherently available to a given melanoma cell-type. Several studies proposed elevated signaling of single RTKs as a mechanism of BRAFi resistance [21–28]. So far, however, the potential role of RTK profiling as a classification tool to discriminate BRAFi-resistant and -susceptible tumors has not been evaluated.

In this study we assessed whether the expression pattern of RTK genes could stratify melanomas in different groups. By integrating the RTK classification with the previously identified MPSE phenotypes [17], we derived a robust classification of melanoma tumors in three subtypes that was validated in both cell lines and clinical samples. This classification led to the identification of a new melanoma subtype displaying intrinsic resistance to targeted therapy against mutant BRAF. Moreover, we provide evidence of PI3K/mTOR signaling pathway dependency of such intrinsically resistant cells.

## RESULTS

### Melanoma subtypes identification in CCLE dataset

**Figure 1:**
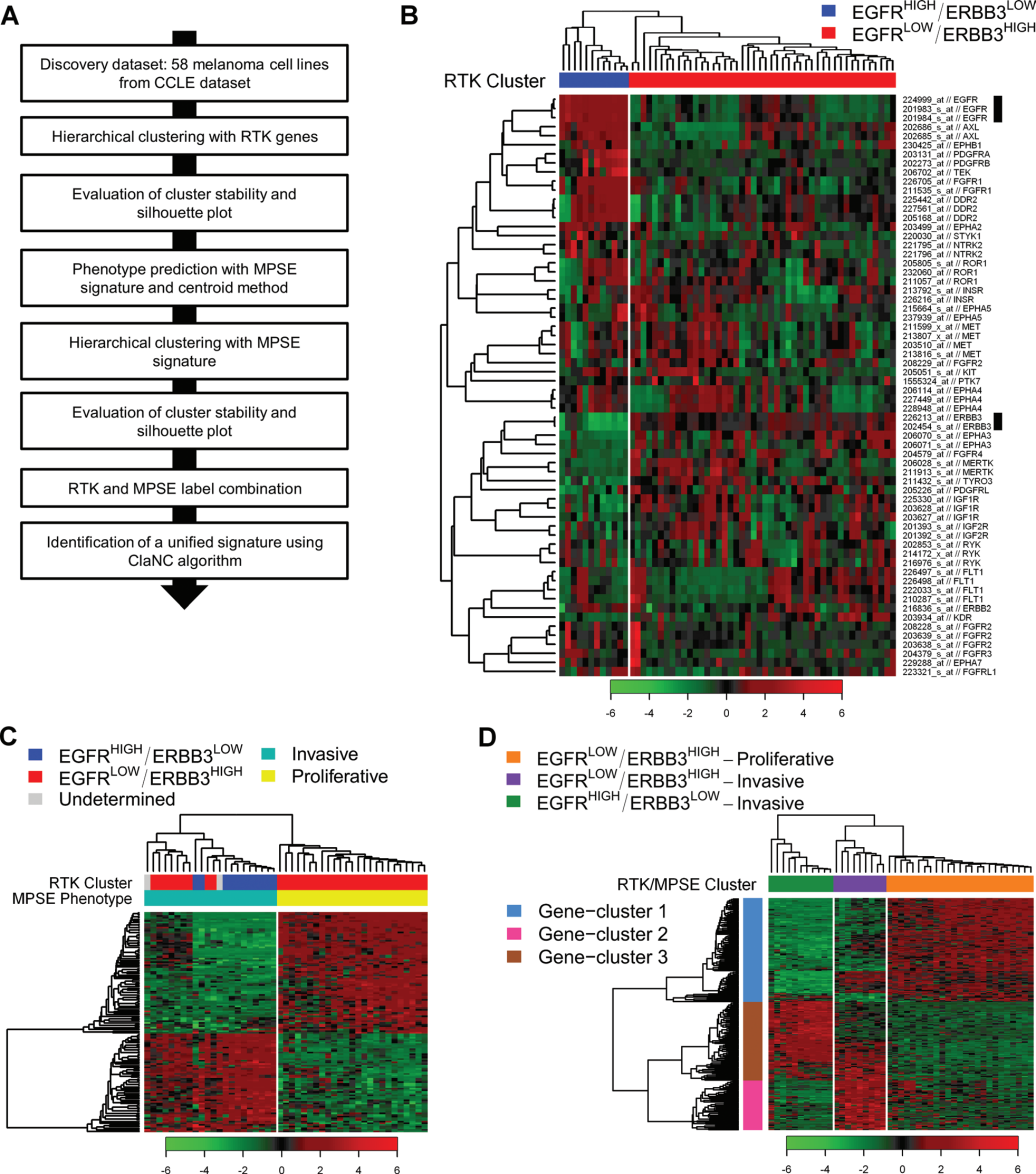
Melanoma subtypes identification by class discovery in the CCLE dataset **(A)** Workflow of bioinformatics analysis. **(B)** RTKs expression of 58 melanoma cell lines grouped by hierarchical clustering. Samples are separated in two major clusters named EGFR^HIGH^/ERBB3^LOW^ (blue) and EGFR^LOW^/ERBB3^HIGH^ (red). Black bars highlight *EGFR* and *ERBB3* probe sets. **(C)** Hierarchically clustered heatmap showing the expression of MPSE genes in the 47 samples with a defined phenotype: light green, Invasive; yellow, Proliferative. RTK labels are shown for comparison. **(D)** Heatmap of the hierarchical clustering using the RTK/MPSE signature: EGFR^HIGH^/ERBB3^LOW^-Invasive (green); EGFR^LOW^/ERBB3^HIGH^-Invasive (violet); EGFR^LOW^/ERBB3^HIGH^-Proliferative (orange). The number of clustered samples, as detailed in the text, is forty-five. The 210 genes of the RTK/MPSE signature are grouped into three highly correlated clusters: 1, light-blue; 2, pink; 3, brown.

We hypothesized that different melanoma subtypes could exist on the basis of the gene expression pattern of RTK genes. To assess our hypothesis we applied a class discovery approach (Figure 1A) to gene expression data of 58 melanoma cell lines (Supplementary Table 1) included in the Cancer Cell Line Encyclopedia (CCLE) [29]. We selected 177 probe sets representing 57 unique RTKs. Based on the gene expression barcode, 64 probe sets, mapping on 34 unique RTKs and expressed in at least 5% of samples, were used to perform hierarchical clustering (HC) (Figure 1B). We found two major clusters composed of 12 and 46 samples and characterized by distinct expression patterns of RTK genes. In particular, the two clusters were marked by mutually exclusive expression of *ERBB3* and *EGFR* (Figure 1B), thus they were named EGFR^HIGH^/ERBB3^LOW^ and EGFR^LOW^/ERBB3^HIGH^. Additionally, consensus hierarchical clustering (CHC) was applied and we observed that HC and CHC were highly concordant in assigning samples to the two subtypes, indicating a good robustness of these classes (Supplementary Figure 1A). Finally, according to silhouette analysis, 56 samples had a positive silhouette score and were representative of their cluster assignment (Supplementary Figure 1B), while two samples with a negative silhouette score were classified as “undetermined”.

We next asked how our newly identified RTK subtypes compared with the invasive and proliferative phenotypes. For this purpose we used the MPSE signature [17] to predict the phenotype of the 58 melanoma cell lines included in CCLE. This prediction assigned 22 samples to the invasive phenotype and 25 to the proliferative one; the remaining 11 cell lines could not be assigned to any phenotype (Supplementary Figure 1C). A clear and robust distinction in two groups of the 47 classified cell lines was confirmed by HC and CHC using the MPSE signature (Figure 1C and Supplementary Figure 1D). Silhouette analysis proved that all samples were correctly classified (Supplementary Figure 1E). Comparing the RTK and MPSE classifications we found that all EGFR^HIGH^/ERBB3^LOW^ samples were included in the invasive phenotype while EGFR^LOW^/ERBB3^HIGH^ samples were invasive or proliferative (Figure 1C). Therefore, we combined RTK and MPSE subtypes leading to the following classification in three main subtypes: EGFR^HIGH^/ERBB3^LOW^-Invasive; EGFR^LOW^/ERBB3^HIGH^-Invasive; EGFR^LOW^/ERBB3^HIGH^-Proliferative. Samples with an undetermined classification by RTK or MPSE were not assigned to any of the three subtypes and were excluded from subsequent analyses.

This new classification was obtained by sequential stratification of samples using two distinct gene lists. To retrieve an integrated gene signature, able to directly discriminate the three subtypes, we applied the ClaNC algorithm and we identified a 210-gene signature (named hereafter RTK/MPSE) (Supplementary Table 2) as the minimal set of genes with lowest classification error rate (error rate in five-fold cross-validation = 0%). Among RTK genes, both *EGFR* and *ERBB3* were included in this signature. The ability of the new RTK/MPSE signature to discriminate the three subtypes was assessed by HC (Figure 1D) and the accuracy of such classification was confirmed. These 210 genes clustered in three major groups of highly correlated genes (Figure 1D and Supplementary Table 2). We performed functional analysis of these gene-clusters to identify biological pathways distinguishing our melanoma subtypes (summarized in Table 1). EGFR^LOW^/ERBB3^HIGH^-Proliferative subtype was defined by *MITF*, MITF-regulated genes and other genes involved in melanocyte development and pigmentation signaling (gene-cluster 1). EGFR^HIGH^/ERBB3^LOW^-Invasive melanomas could be distinguished from EGFR^LOW^/ERBB3^HIGH^-Invasive by down-regulation of genes involved in antigen presentation pathways and coding for HLA class II antigens and their transcriptional regulators (gene-cluster 2) and for higher expression of genes associated to regulation of epithelial-mesenchymal transition, cellular movement, constituent of extracellular matrix and growth factors (gene-cluster 3). These later genes were down-regulated in the EGFR^LOW^/ERBB3^HIGH^-Proliferative subtype. These results indicate that expression of genes involved in relevant biological pathways such as melanoma development, differentiation, immunity and metastatic behavior allows dissection of melanoma heterogeneity in three clearly separated subtypes. Furthermore, this classification led to the discovery of the EGFR^HIGH^/ERBB3^LOW^-Invasive subtype, previously hidden in the broader phenotypic class of invasive tumors.

**Table 1:** Summary of the main features of RTK/MPSE subtypes Upwards arrows represent activation or high expression, downwards arrows inhibition or low expression. The number of arrows indicate the strenght of the feature

	EGFR^HIGH^/ERBB3^LOW^-Invasive	EGFR^LOW^/ERBB3^HIGH^-Invasive	EGFR^LOW^/ERBB3^HIGH^-Proliferative
**Subtype frequency [n, (%)] in**			
CCLE	11 (24.4)	9 (20.0)	25 (55.6)
Meta-Cell	25 (14.1)	25 (14.1)	127 (71.8)
Meta-Clinical	46 (12.8)	36 (10.0)	277 (77.2)
**Pathways defined by RTK/MPSE genes (activation or inhibition)**			
Eumelanin biosynthesis	↓↓	↓	↑↑
Melanocyte development and pigmentation signaling	↓↓	↓	↑↑
Antigen presentation	↓↓	↑↑	↓
Allograft rejection signaling	↓↓	↑↑	↓
OX40 signaling	↓↓	↑↑	↓
Hepatic fibrosis / hepatic stellate cell activation	↑↑	↑	↓↓
Human embryonic stem cell pluripotency	↑↑	↑	↓↓
Regulation of the epithelial-mesenchymal transition	↑↑	↑	↓↓
**RTKs expression (high or low expression)**			
AXL	↑↑	↑↑	↓↓
EGFR	↑↑	↓	↓↓
EPHA2	↑	↑	↓
ERBB3	↓↓	↑↑	↑↑
MERTK	↓↓	↓↓	↑
PDGFRA	↑	↓	↓
PDGFRB	↑	↓	↓
**PLX4720 sensitivity**			
Resistant	✓	✓	
Intermediate		✓	✓
Sensitive		✓	✓

### Subtypes validation in independent datasets

**Figure 2:**
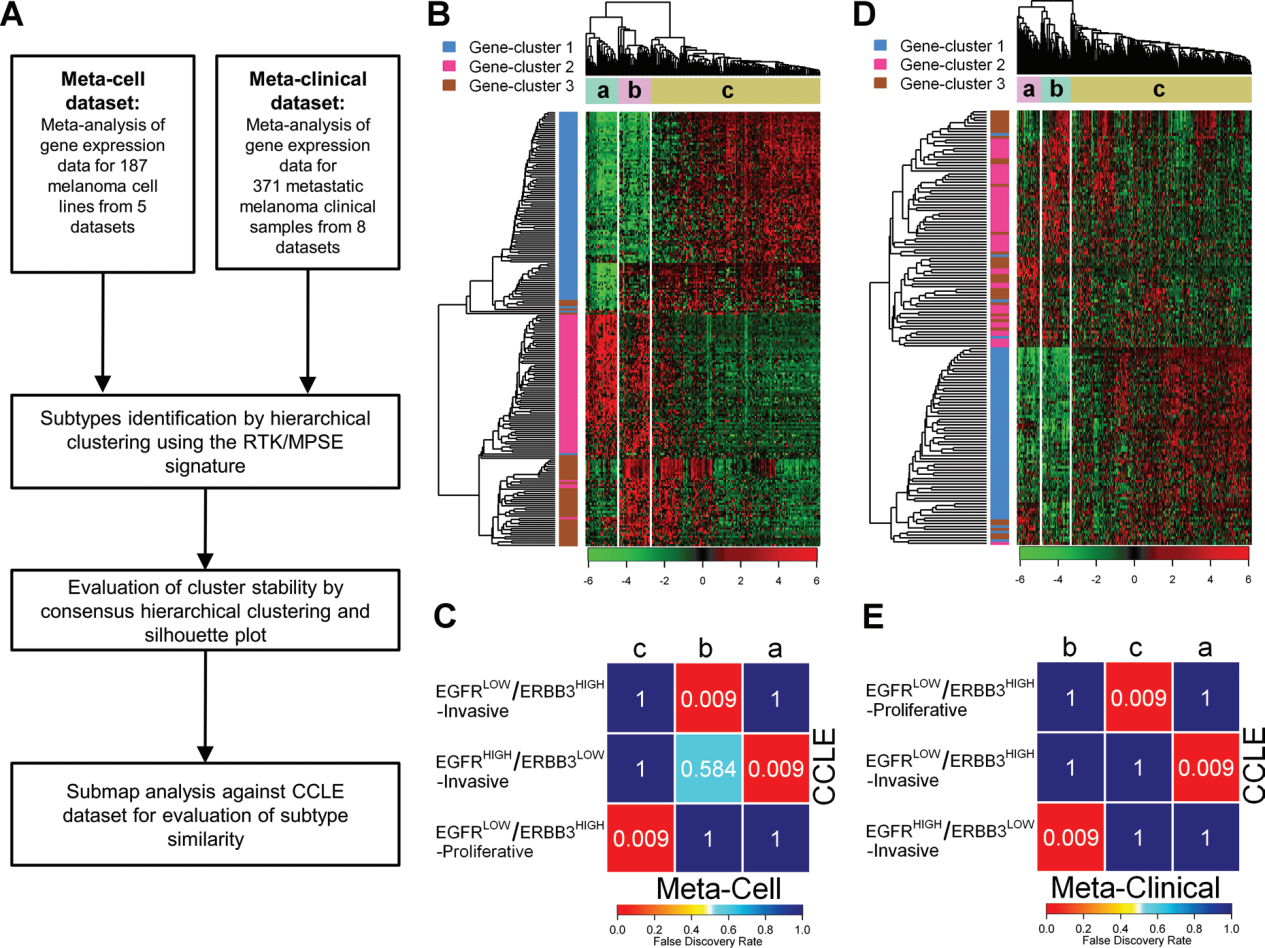
Subtypes validation in independent datasets **(A)** Workflow of bioinformatic subtype validation. **(B)** and **(D)** Heatmap of hierarchical clustering of Meta-Cell (B) and Meta-Clinical (D) datasets according to the RTK/MPSE signature. In each dataset three clusters, indicated with letters a, b, c, were identified. **(C)** and **(E)** SubMap analysis for similarity assessment between clusters identified in Meta-Cell (C) and Meta-Clinical (E) and CCLE subtypes. The colors of the heatmap represent the false discovery rate, reported also numerically, which measure the similarity of the subtypes. Meta-Cell and CCLE datasets had 13 cell lines in common: 9 were assigned to the same subtype, 3 had an undetermined classification in one of the two datasets and 1 had a discordant classification.

To assess the reproducibility of the three melanoma subtypes identified in CCLE, we collected gene expression data for 187 melanoma cell lines from five different studies (Supplementary Table 1) and integrated them in a meta-analysis dataset (Meta-Cell dataset) using ComBat to correct for inter-study batch effects (Supplementary Figures 2A–2B). The process of subtype validation is summarized in Figure 2A. By HC and CHC using the RTK/MPSE signature, Meta-Cell dataset was divided in three robust clusters (Figure 2B and Supplementary Figure 3A). A positive silhouette width was observed for 95% of samples (Supplementary Figure 3B). The genes contained in the RTK/MPSE signature conserved their correlation patterns as 197/210 genes fell into the previously defined gene-clusters, indicating a similarity between melanoma subtypes defined in CCLE and Meta-Cell datasets (Figure 2B). SubMap analysis confirmed a univocal correspondence between subtypes identified in the two independent datasets (Figure 2C).

The same approach was applied to gene expression data of 371 metastatic melanoma tissue samples collected from 8 public datasets (Meta-Clinical dataset, Supplementary Table 1). ComBat adjustment efficiently removed the inter-study technical variation that was inflated by the merging of datasets profiled with different platforms (Supplementary Figures 2C–2D). Despite only 154 out of 210 genes of the RTK/MPSE signature were available in Meta-Clinical dataset, HC and CHC revealed a robust separation of patients in three robust clusters (Figure 2D and Supplementary Figure 3C). The majority (97%) of samples were correctly assigned to their cluster membership as they had a positive silhouette width (Supplementary Figure 3D). SubMap analysis revealed that the clinical subtypes were identical to those identified in CCLE (Figure 2E).

Overall our RTK/MPSE signature enables the identification of distinct biological melanoma subtypes in a robust and reproducible manner, not only in cell lines but also in metastatic melanoma clinical samples.

### RTK expression patterns in melanoma subtypes

**Figure 3:**
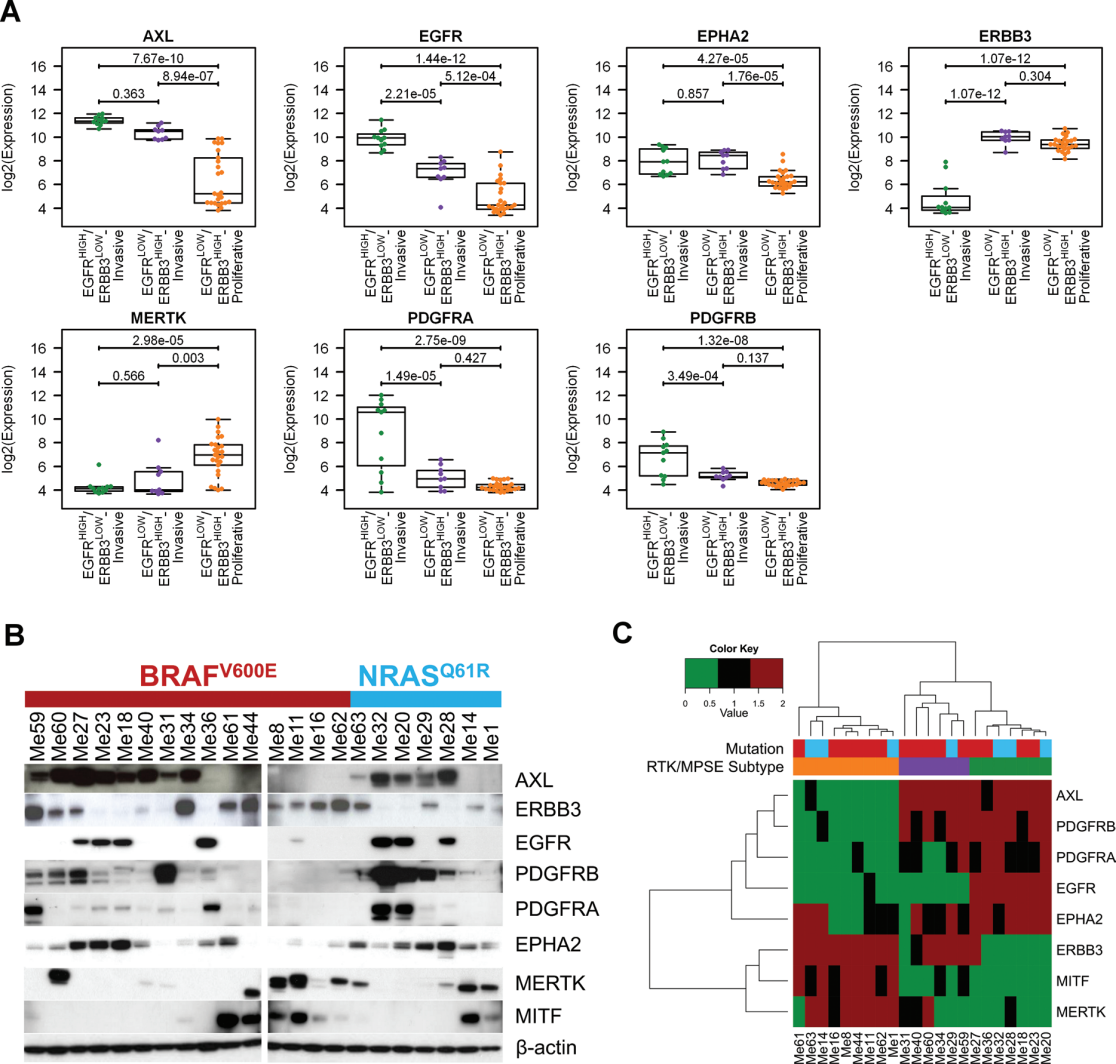
Expression pattern of selected RTKs in melanoma subtypes **(A)** Box-plot showing the distribution of log2 expression values of RTKs with the most significant differential expression according to melanoma subtypes in CCLE dataset. Statistical analysis by ANOVA followed by Tukey's post-hoc test. **(B)** Western blot analysis of the seven most relevant RTKs and MITF in the panel of 22 melanoma cell lines. The BRAF and NRAS mutational status is reported. Since preliminary results indicated that levels of RTKs were not affected by culture conditions such as serum-free or serum-containing medium, all analyses were performed by cells grown under standard culture conditions. **(C)** Heatmap representing the hierarchical clustering of protein expression investigated in panel (B) for 22 melanoma cell lines. The RTK/MPSE subtype and mutational status are reported and are colored as in (A) and (B) respectively.

Differential expression analysis was carried out using ANOVA to identify, between the 34 RTKs, those with subtype-specific gene expression patterns in both CCLE and Meta-Cell datasets (Supplementary Table 3). We found 30 RTKs differentially expressed at a false discovery rate (FDR) < 0.05 in at least one of the two cell line datasets. However, to focus on the most relevant ones we selected seven genes (*AXL*, *EGFR*, *EPHA2*, *ERBB3*, *MERTK*, *PDGFRA* and *PDGFRB*) with an FDR < 0.0001 and a fold-change ≥ 2.5 in at least one of the contrasts, in both datasets. Their expression levels according to subtypes are shown in Figure 3A for CCLE. The same analysis was repeated for the Meta-Clinical dataset and the differential expression of 6 of the 7 RTKs was validated (FDR < 0.05, Supplementary Table 3), except for *EPHA2*, which however showed a trend of differential expression (FDR = 0.052). The fold-changes between contrasts observed in Meta-Clinical dataset were lower compared to cell lines but gene expression data of clinical samples could be affected by a higher noise, due to intra-tumor heterogeneity and merging of multiple microarray platforms.

We then examined the protein expression of these seven RTKs and *MITF* in a panel of 22 patient-derived melanoma cell lines (Supplementary Table 4) by Western blot analysis (Figure 3B). With a few exceptions, the protein expression patterns in these cell lines reflected the observations made at the gene level in the three subtypes: EGFR and ERBB3 had a mutually exclusive expression and all samples with high expression of EGFR and absent/low of ERBB3 were a subset of the AXL-positive/MITF-negative (Invasive) ones. The band intensities for each protein were transformed to a numerical score corresponding to absent (0), low (1) and intermediate-high (2) expression levels. According to HC of these Western blot scores, samples were clustered in three distinct groups, whose RTK expression pattern resembled EGFR^HIGH^/ERBB3^LOW^-Invasive, EGFR^LOW^/ERBB3^HIGH^-Invasive and EGFR^LOW^/ERBB3^HIGH^-Proliferative subtypes (Figure 3C). Western blot analysis of additional RTKs showed that ERBB2, DDR1, DDR2 and IGF1R were expressed by the majority, but not all cell lines and that MET and KIT displayed a more restricted distribution (Supplementary Figure 4) highlighting further level of complexity within each subtype.

### Drug resistance of melanoma cell lines according to subtypes

**Figure 4:**
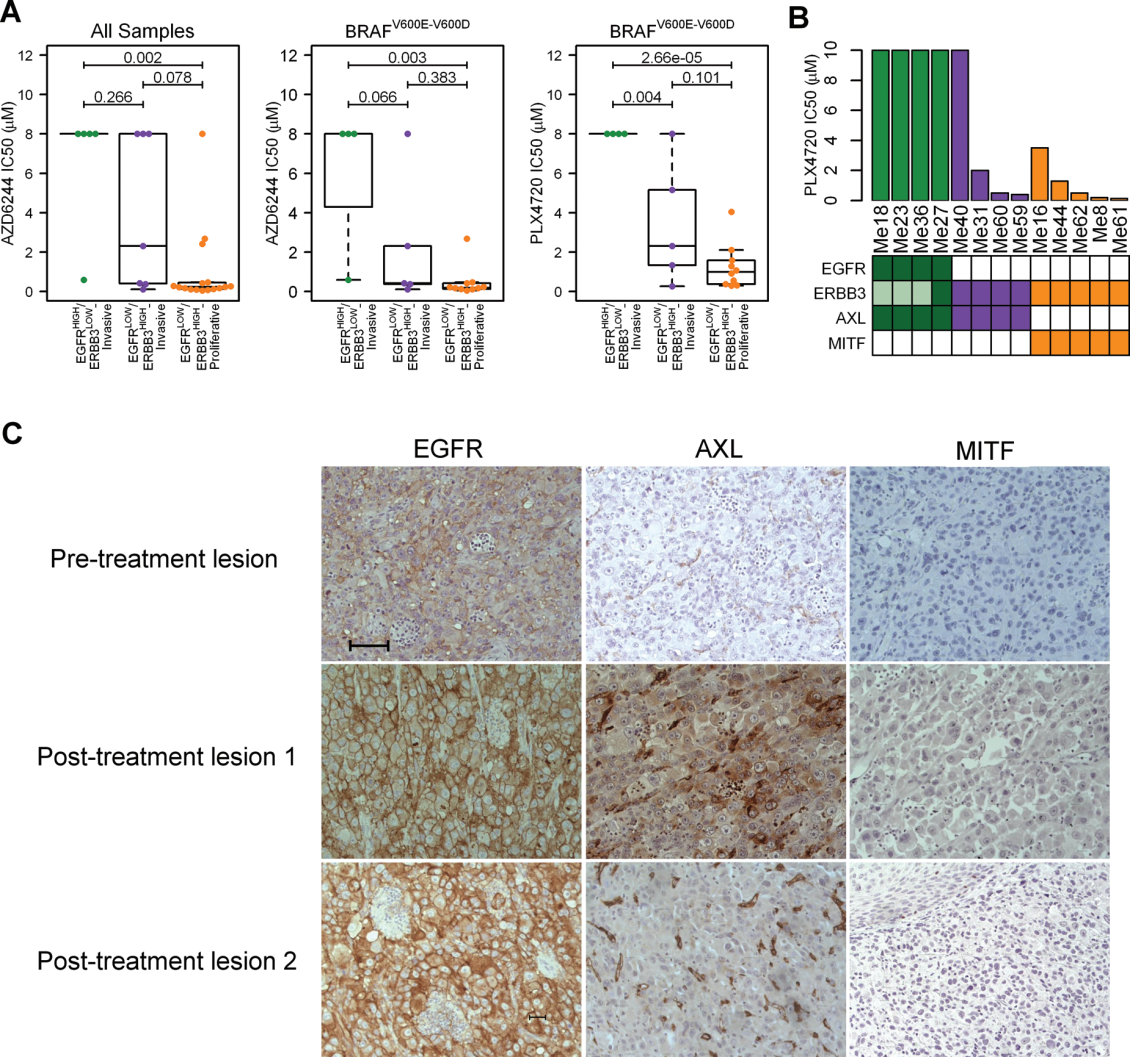
Identification of the EGFR^HIGH^/ERBB3^LOW^-Invasive melanoma subtype as intrinsically resistant to BRAFi **(A)** Box-plot displaying the distribution of IC50 values for AZD6244 and PLX4720 in CCLE dataset according to RTK/MPSE subtypes. For AZD6244, data considering all samples (left panel) or BRAF mutated only (middle panel) are shown. For PLX4720, only samples with BRAF mutations (V600E or V600D) were selected (right panel). Statistical analysis by ANOVA followed by Tukey's post-hoc test. **(B)** PLX4720 IC50 values of BRAF^V600E^ melanoma cell lines representative of the different subtypes (green: EGFR^HIGH^/ERBB3^LOW^-Invasive; violet: EGFR^LOW^/ERBB3^HIGH^-Invasive; orange: EGFR^LOW^/ERBB3^HIGH^-Proliferative). Expression of EGFR, ERBB3, AXL and MITF is summarized from Figure 3B. Presence of the corresponding protein is indicated by full colored boxes; absence as empty box. For ERBB3, shaded boxes indicate low expression. **(C)** EGFR, AXL and MITF staining in serial sections of pre- and post-treatment melanoma specimens from a patient intrinsically resistant to Vemurafenib treatment (Patient P3). 20x magnification. Scale bar = 100 μm.

To determine whether our classification could allow prediction of melanoma response to drugs, we evaluated by ANOVA how melanoma subtypes correlated with drug-sensitivity data (IC50) in CCLE (Supplementary Table 5). When all melanoma cell lines (*n* = 28) were considered, the three subtypes showed a significantly different response (FDR < 0.05) to the MEK inhibitor AZD6244 (Figure 4A), to the SRC family kinase (SFK)/ABL dual-kinase inhibitor AZD0530 and to the ALK inhibitor TAE684 (Supplementary Table 5). Considering BRAF-mutant tumors only (V600E or V600D, *n* = 19), subtype-specific response was still observed for AZD6244 (FDR = 0.030) (Figure 4A) and, in addition, for PD-035901 (FDR = 0.030), a second MEK inhibitor (Supplementary Table 5). The most striking drug-response difference was however observed for PLX4720 (FDR = 0.001) with EGFR^HIGH^/ERBB3^LOW^-Invasive being the most resistant (Figure 4A). Pearson's correlation between gene expression CCLE data and IC50 of PLX4720 across BRAF-mutated melanomas confirmed that all seven RTK genes were directly (*EGFR*, *AXL*, *EPHA2*, *PDGFRA* and *PDGFRB*; *r* between 0.6 and 0.8) or inversely correlated (*MERTK* and *ERBB3*; *r* between –0.4 and –0.6) to PLX4720 resistance. *EGFR* was the first RTK and the seventh most positively correlated gene (*r* = 0.845; *p*-value = 5.34e-06). This differential sensitivity to PLX4720 treatment was validated *in vitro* in our panel of patient-derived melanoma cell lines after treatment with increasing concentrations of PLX4720 (Figure 4B). As in CCLE, EGFR^HIGH^/ERBB3^LOW^-Invasive cells were all highly resistant to PLX4720 (IC50 > 10 μM, taken as the maximum value), EGFR^LOW^/ERBB3^HIGH^-Proliferative were sensitive or moderately resistant. The AXL-positive EGFR^LOW^/ERBB3^HIGH^-Invasive subtype showed the same heterogeneous sensitivity observed in CCLE, with one cell line being highly resistant and the others sensitive or moderately resistant.

The clinical significance of EGFR expression was examined in ten surgical specimens of five patients with BRAF-mutant melanomas treated at our Institute with the BRAFi Vemurafenib (Supplementary Table 6). The pre-therapy lesion of patient P3, one of the three patients intrinsically resistant to therapy and rapidly progressing under treatment, already displayed strong EGFR staining. A clear increase in intensity was also found in two progressing metastases taken after therapy (Figure 4C). None of these specimens expressed MITF, whereas one of the two progressing lesion co-expressed AXL (Figure 4C). Pre- or post-therapy melanoma samples from the other two unresponsive patients (P2 and P5) and from the two responsive ones (P1 and P4), instead, did not have detectable staining of EGFR or AXL. All together these data indicates that mRNA and protein abundance of a core group of RTK are distinguishing traits for the classification of melanoma subtypes. Furthermore the RTK/MPSE signature allows identification of BRAF tumors intrinsically resistant to BRAFi with higher accuracy in comparison to the *AXL/MITF* dual transcriptional state. Similarities and differences of the three melanoma subtypes are summarized in Table 1.

### PI3K/mTOR signaling pathway dependency of PLX4720-resistant EGFR^HIGH^/ERBB3^LOW^-Invasive melanoma cells

**Figure 5:**
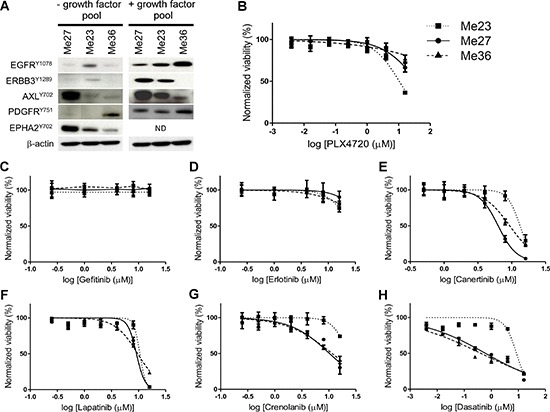
RTK activation and effect of RTKs inhibitors on BRAF^V600E^ EGFR^HIGH^/ERBB3^LOW^-Invasive melanoma cell lines **(A)** Basal RTK and ligand induced phosphorylation. Melanoma cell lines were cultured overnight in the absence of serum and stimulated for 10 minutes with serum-free medium or with medium containing a pool of growth factors: EGF, NRG1-β1/HRG1-β1, PDGF (all at 100 ng/ml) and GAS6 500 ng/ml. All RTKs were basally phosphorylated, although at different level, in Me27; all, except PDGFR, in Me23; all, except PDGFR and ERBB3, in Me36. Ligand stimulation increased or induced phosphorylation of all RTKs with the exception of ERBB3 for Me36. **(B–H)** Representative growth curves, based on alamarBlue viability assays, of Me23, Me27 and Me 36 following a 72 h treatment with the indicated inhibitor. Means and standard deviations of three replicates are shown. Normalized viability (%) is relative to vehicle-treated (DMSO) control cells.

To explore whether signaling pathway dependencies could be similar in the BRAFi intrinsically resistant EGFR^HIGH^/ERBB3^LOW^-Invasive samples, three out of the four BRAF^V600E^ melanoma cell lines representative of this subtype were chosen. Me23 and Me36 had high expression of EGFR, negligible expression of ERBB3 and expressed AXL at different levels. Although grouped in the same cluster, Me27 was unique since it co-expressed EGFR, AXL and ERBB3. Western blot analysis indicated presence of basally phosphorylated (p) EGFR, AXL and EPHA2 in all three cell lines (Figure 5A), whereas pPDGFRB was detectable in Me36 and pERBB3 in Me23. Me27 displayed a faint band in correspondence of pPDGFRB and pERBB3. In each cell lines, stimulation by a pool of growth factors specific for these RTKs increased or induced ex novo phosphorylation of all RTKs except ERBB3 for Me36 (Figure 5A). These cell lines were all resistant to PLX4720 (Figure 5B).

**Figure 6:**
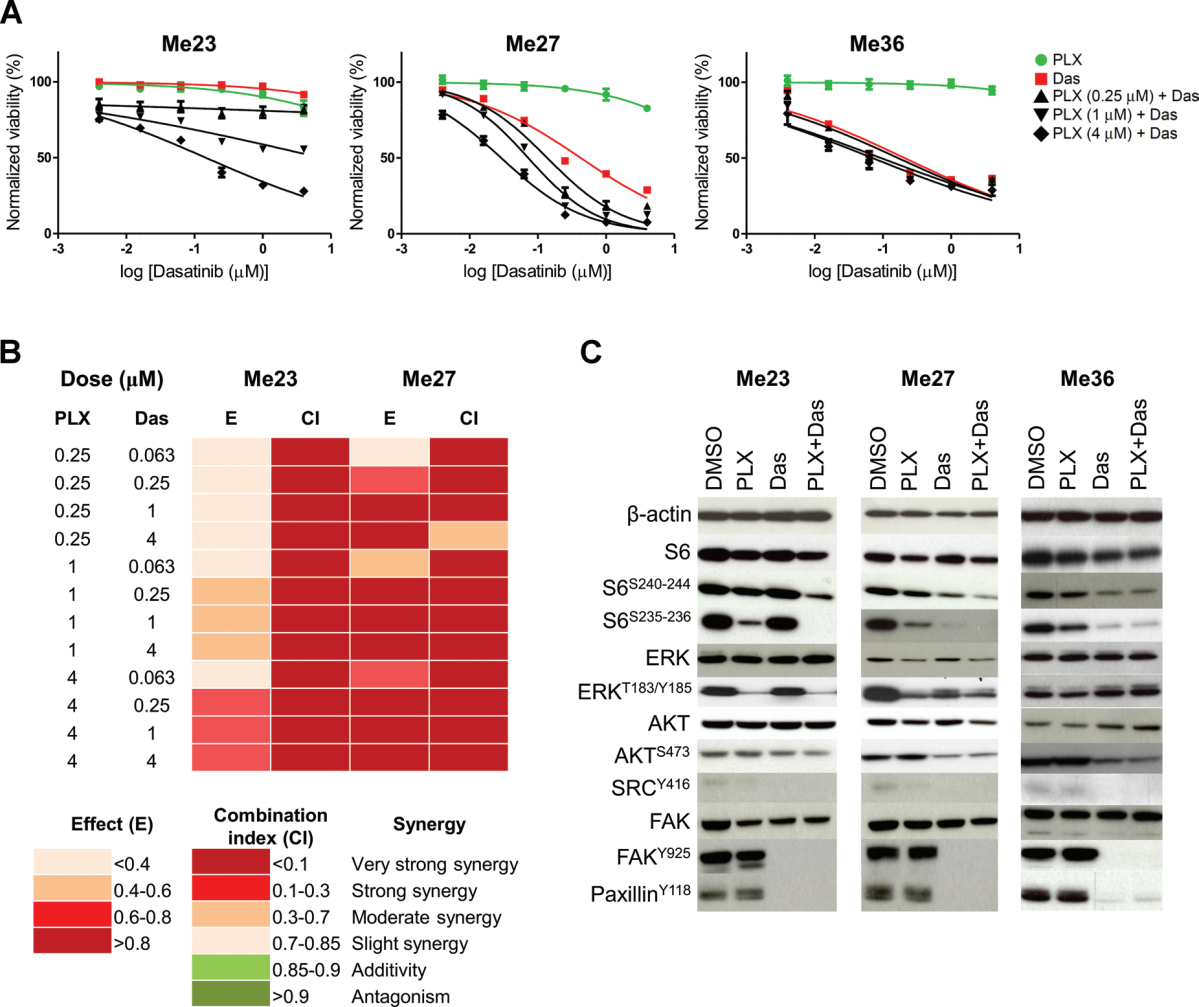
Effect of Dasatinib and Dasatinib/PLX4720 combination on BRAF^V600E^ EGFR^HIGH^/ERBB3^LOW^-Invasive melanoma cell lines **(A)** Dose-response curves to Dasatinib, PLX4720 and their combination. Dasatinib IC50 was 8.54 μM for Me23, 0.6 μM for Me27 and 0.41 μM for Me36. Data shown are means and standard deviations of three replicates. **(B)** Drug interaction analysis by Chou and Talalay method. Shown are color-coded fraction affected values (effect, E) and combination indexes (CI) for Me23 and Me27 at the indicated drug combination doses. The color-code is shown at the bottom of the panel. **(C)** Effects of a 24 h treatment with PLX4720 (PLX, 1 μM), Dasatinib (Das, 0.25 μM) and their combination (PLX+Das) on phosphorylation of signaling molecules in Me23, Me27 and Me36. The combination between PLX4720 and Dasatinib reduced phosphorylation of S6 (S235–236 and S240–244) in comparison to single drugs when growth inhibitory effect was synergistic (Me23 and Me27). Controls are vehicle (DMSO) treated cells.

Pharmacologic blockade of EGFR signaling by Gefitinib and Erlotinib was, however, unable to affect growth of these cell lines (Figures 5C–5D). Signaling blockade of ErbB family (EGFR, HER2, ERBB3) by Canertinib, of EGFR and HER2 by Lapatinib and of PDGFR by Crenolanib could be effective only at high dose (Figures 5E–5G). None of these drugs, with the exception of Caneternib and Crenolanib on Me23, showed a synergistic effect with PLX4720 (data not shown). Two out of the three melanoma lines (Me27 and Me36) were instead sensitive, at low μM dose, to Dasatinib, a multikinase inhibitor [30] (Figure 5H). In addition, the PLX4720/Dasatinib combination was able to reduce growth of Me23 and to further enhance cell growth inhibition of Me27, in comparison to both drugs alone (Figure 6A) and the effect was strongly synergistic (Figure 6B). To identify the signaling pathway/s inhibited by the combination of PLX4720 and Dasatinib we examined, by Western blot, the activation of components of PI3K/AKT/mTOR, ERK/MAPK and SRC/FAK/Paxillin, following single or combined drug treatments (Figure 6C). Overall, we observed that the PLX4720/Dasatinib combination was more effective in abrogating or reducing, in Me23 and Me27, levels of phosphorylated (p) ribosomal protein S6, a marker of mammalian target of rapamycin complex 1 (TORC1) activity [31], at both pS6^S235–236^ and pS6^S240–242^ sites. The effect of the drug combination in comparison to single drugs was instead similar for Me36 that displayed similarly sensitivity to Dasatinib and PLX4720/Dasatinib combination (Figure 6C). The effect of Dasatinib alone on pS6 was also associated to the degree of Dasatinib sensitivity (Figure 6C). All together, these findings suggested a convergence of the combined treatment on suppression of TORC1 activity. As expected, Dasatinib completely abrogated pSRC and downstream targets, pPaxillin and pFAK in all three cell lines and reduced pAKT levels in Me27 and Me36. PLX4720 reduced pERK in Me23 and Me27. The PLX4720/Dasatinib combination was unable to modulate these molecules any further, indicating that they were not a necessary requirement to allow a synergistic growth inhibitory effect to occur.

**Figure 7:**
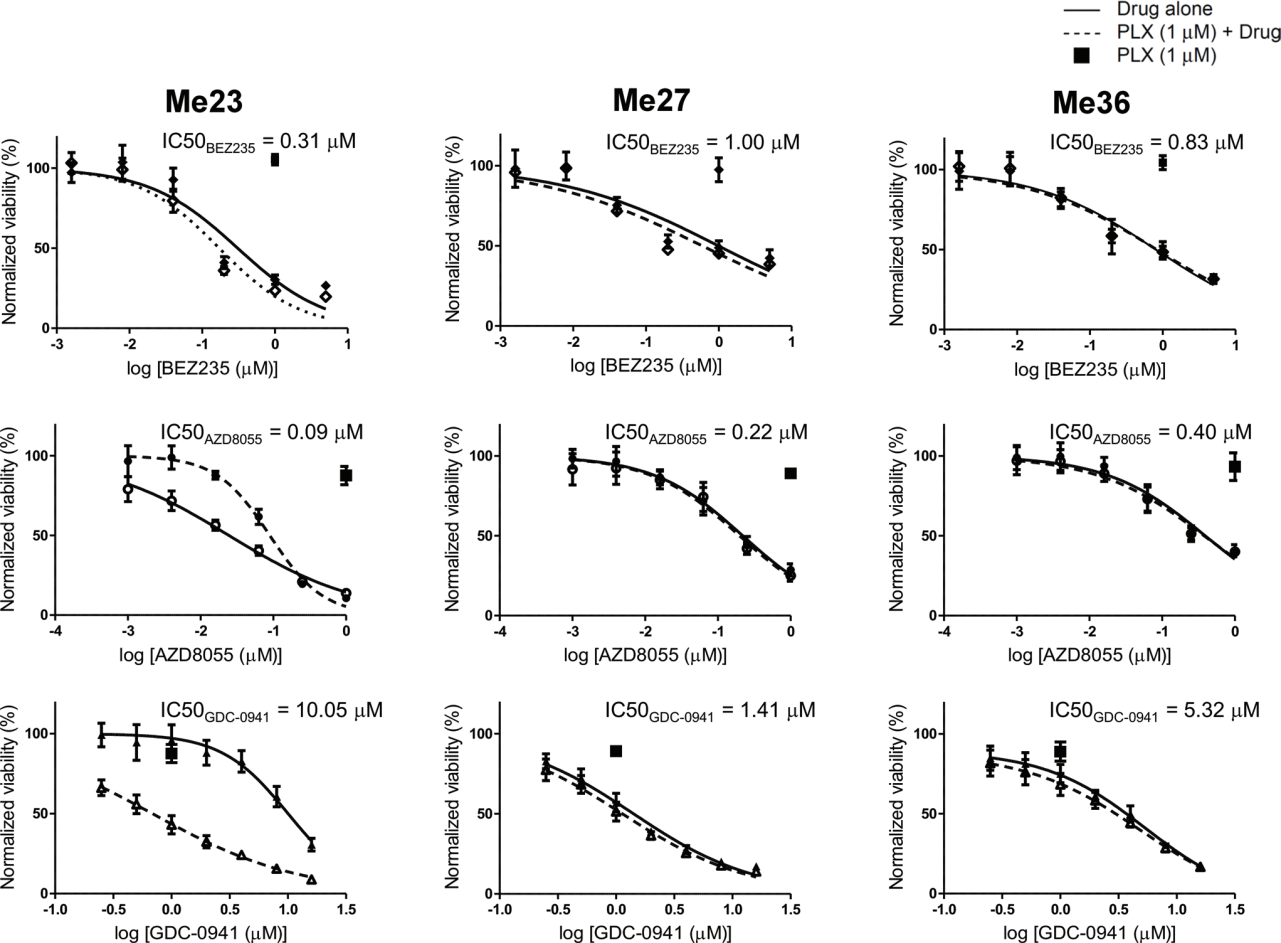
PI3K/mTOR inhibitors reduce viability of BRAF^V600E^ EGFR^HIGH^/ERBB3^LOW^-Invasive melanoma cell lines Dose-response curves of Me23, Me27 and Me36 to BEZ235, GDC-0941, AZD8055 alone or in combination with PLX4720 (1 μM). Data shown are means and standard deviations of three replicates. Normalized viability (%) is relative to vehicle-treated (DMSO) controls. For each cell line, IC50 values of PI3K and/or mTOR inhibitors are listed above each graph.

To prove that PI3K/mTOR signaling inhibition could be relevant for these intrinsically resistant cells, we used BEZ235, a selective inhibitor of the class I phosphoinositide 3-kinase (PI3K) enzymes and mTORC1 and 2, GDC-0941, a PI3K inhibitor (PI3Kα/δ), and AZD8055, an inhibitor of mTORC1 and 2. BEZ235 and AZD8055 potently inhibited the *in vitro* growth of all three melanoma cell lines (Figure 7A and 7B), whereas GDC-0941 was less potent (Figure 7C). Inhibition of RAF/MAPK/ERK pathway by PLX4720 exerted no further effect on cell growth of two out of three cell lines. Only on Me23, the combination of PLX4720 with GDC-0941, or AZD8055 achieved synergistic growth inhibitory effect indicating that, for this particular melanoma, concurrent suppression of RAF/MAPK/ERK pathway is necessary to affect growth when only PI3K or mTOR signaling is abrogated. Taken together, these results indicate that blockade of upstream RTKs can achieve only modest growth inhibitory activity in PLX4720-resistant cells of the EGFR^HIGH^/ERBB3^LOW^-Invasive subtype, whereas their growth could be affected when both PI3K and mTORC1/2 are effectively targeted, even without simultaneous inhibition of RAF/MAPK/ERK pathway.

## DISCUSSION

In this study, we explored whether expression levels and types of RTKs, frequently identified as targets of negative feedback loop release and involved in drug resistance mechanisms [21–28, 32–34], could identify subtypes differing in terms of sensitivity to BRAFi. Hierarchical clustering was therefore used to examine RTK gene expression similarities among a high number of samples (245 melanoma cell lines and 371 clinical samples). Taking into account the previously described MPSE phenotypes [17], we identified three stable melanoma subtypes. Our classification significantly improves detection of intrinsically resistant melanoma cells. Indeed, melanomas belonging to one of the two previously described divergent phenotypes [12–17], displaying high levels of *AXL*, were recently shown to exhibit intrinsic resistance to MAPK-pathway inhibitors [20]. However, as shown in CCLE dataset and by drug sensitivity assays on melanoma cell lines, melanomas with high expression of *AXL* include both resistant and sensitive tumors. Instead, our newly defined EGFR^HIGH^/ERBB3^LOW^-Invasive subtype is composed only of resistant tumors.

EGFR has been involved in adaptive and acquired resistance of melanoma to BRAFi. In one study, 6 post-treatment biopsies from 16 patients who developed resistance to BRAFi or MEKi expressed EGFR [28]; in another, EGFR was hyperphosphorylated in 4 post-therapy lesions from 5 relapsed patients [23]. The first study hypothesized that increased EGFR could be the result of an adaptive response of melanoma cells occurring in presence of the drug and due to the activation of TGFβ signaling following loss of the transcription factor SOX10 [28]. Knockdown of *SOX10* led to increased EGFR expression and to a concomitant decrease of MITF [28]. We here describe that these features, expression of *EGFR*, lack of *MITF* and *SOX10*, are inherent and occurs independently of drug selection in EGFR^HIGH^/ERBB3^LOW^-Invasive melanomas. Both *MITF* and *SOX10* are included in the RTK/MPSE signature (cluster 1) and are down-regulated in this subtype. Our *in vivo* evidence of EGFR expression in both pre-therapy and post-therapy lesions of one out of three unresponsive patients, highlight the need to assess expression of EGFR and other RTKs in pre-and post-therapy lesions of a larger cohort of BRAF-mutant patients with different response to BRAFi treatment. These studies will inform on the value of EGFR and other RTKs as biomarkers to identify BRAFi intrinsically resistant tumors and will allow evaluation of their frequency at all stages of resistance from intrinsic to adaptive and acquired.

In some instances but not in others, mechanisms of resistance involving RTKs can be counteracted by inhibition of RTKs often cooperating with PLX4720 to inhibit growth of resistant cells *in vitro* and *in vivo* [20, 23, 27, 28]. In our three melanoma cell lines representing the PLX4720-resistant EGFR^HIGH^/ERBB3^LOW^-Invasive subtype, inhibition of single RTKs had limited efficacy alone or in combination with PLX4720. Among the drugs tested, only Dasatinib was effective and combination of PLX4720 with Dasatinib resulted in a synergistic shifting of the dose response even in Me23, which was resistant to both single drugs. Dasatinib has a wide kinase profile and targets PDGFRB, EPHA2, KIT, DDR1 and DDR2, in addition to SRC family kinases and BCR–ABL [30]. Inhibition of these RTKs may be particularly relevant to counteract the release of negative feedback loops with the consequent activation of various signaling proteins [32–34]. The synergy between PLX4720 and Dasatinib converged on suppression of pS6, a marker mTORC1 activity. Combined inhibition of PI3K and MTORC1/2, an attractive pathway target in melanoma [35], by BEZ235, even without the addition of PLX4720, achieved strong *in vitro* inhibition in these intrinsically resistant cells.

In conclusion, our results describe molecular features allowing identification of a melanoma subtype intrinsically resistant to BRAFi. Further characterization of this subtype may allow definition of a set of biomarkers that, together with RTKs, will aid the prediction of intrinsic resistance. In addition, this study highlights the need to understand signaling networks that, in addition to the one here identified, could guide the choice of effective treatments for this subtype of unresponsive patients.

## MATERIALS AND METHODS

### Gene expression data

The discovery dataset was built starting from the Cancer Cell Line Encyclopedia (CCLE) [29]: raw CEL files (Affymetrix U133 Plus 2.0 array) for 58 melanoma cell lines were obtained from the CCLE database (http://www.broadinstitute.org/ccle/home); mutational status of BRAF was retrieved from CCLE_hybrid_capture1650_hg19_NoCommonSNPs_NoNeutralVariants_CDS_2012.05.07.maf file; drug sensitivity data were extracted from CCLE_NP24.2009_Drug_data_2012.02.20.csv file.

The Meta-Cell dataset was composed of five studies [13, 36–39] profiled with Affymetrix U133 Plus 2.0 array, for a total of 188 melanoma cell lines. The Meta-Clinical dataset was composed of eight studies [40–45] for a total of 378 metastatic melanoma samples profiled with five different platforms. Raw data were downloaded from NCBI Gene Expression Omnibus database [46] (GEO, http://www.ncbi.nlm.nih.gov/geo/).

### Gene expression data processing

For Affymetrix datasets, raw data were pre-processed using frozen RMA [47] as implemented in the frma package of Bioconductor [48]. To distinguish expressed and silenced probe sets we applied the barcode [49] function of frma package. For Illumina datasets, raw data were log_2_ transformed and normalized using the robust spline normalization method implemented in the lumi package [50]. Quality Control was applied to each dataset independently on raw and normalized data. Independent datasets composing the Meta-Cell and Meta-Clinical datasets were normalized separately and merged using ComBat [51]. For identification of the RTK/MPSE signature and for statistical analysis all datasets were collapsed at the gene level selecting the probe with highest variability according to inter-quartile range.

### Identification of melanoma subtypes and subtypes-specific gene signature

Identification of sample clusters according to RTKs expression profiles was achieved using agglomerative hierarchical clustering with average linkage and 1-Pearson's correlation coefficient as distance measure. Cluster stability was determined by consensus hierarchical clustering [52], with average linkage, 1-Pearson's correlation as distance and a sub-sampling ratio of features of 0.8 for 1000 iterations. Silhouette width [53] was calculated to identify representative samples for each class. Phenotype prediction essentially followed the described classification method [17] except for Pearson's correlation coefficient (r) cut-off:

$$\begin{array}{l} \text{Invasive: }{\text{r}}_{\text{inv}}\geq 0.4\text{ and }|{\text{r}}_{\text{inv}}-{\text{r}}_{\text{prol}}|\geq 0.4\\ \text{Proliferative: }{\text{r}}_{\text{prol}}\geq 0.4\text{ and }|{\text{r}}_{\text{inv}}-{\text{r}}_{\text{prol}}|\geq 0.4\\ \text{Undetermined: }{\text{r}}_{\text{prol}}\;<\;0.4\text{ and }{\text{r}}_{\text{inv}}\;<\;0.4\text{ or}\\ |{\text{r}}_{\text{inv}}-{\text{r}}_{\text{prol}}|\geq 0.4 \end{array}$$

ClaNC algorithm [54] was used to select discriminating genes among melanoma subtypes. The number of genes tested for each class varied from 1 to 200 and the optimal gene signature was selected as that with the lowest number of genes and lowest error rate, assessed by five-fold cross-validation. SubClass mapping [55], implemented in the SubMap module version 3 of GenePattern software [56], was applied to assess the similarity between subtypes in discovery and validation datasets. Functional analysis was performed by IPA (Ingenuity^®^ Systems, http://www.ingenuity.com).

### Cell lines

The study was conducted according to Declaration of Helsinki Principles and following Institutional guidelines. All primary and metastatic melanoma cell lines were established *in vitro* from surgical specimens of cutaneous malignant melanomas; their origin, mutational status and membership to molecular subtypes identified in this study, are listed in Supplementary Table 4. Molecular and biological features of these cell lines and methods for identification of mutations in BRAF, NRAS, PTEN and p53 genes and for cell maintenance have been previously reported [19, 57]. Identity of the cell lines was verified by short tandem repeat profiling from early passage cells which are maintained as frozen stocks and used for the experiments. All lines were tested for the absence of Mycoplasma contamination by Hoechst 33258 (Sigma-Aldrich, St. Louis, USA).

### Inhibitors

PLX4720, AZD8055, GDC0941, BEZ235, Canertinib, Erlotinib (Tarceva), Gefitinib (Iressa), Crenolanib, Lapatinib (Tykerb) and Dasatinib (Sprycel) (all from Selleck Chemicals, Houston, TX, USA) were diluted in DMSO at 10 mM/ml and stored at −20°C until use; when added to cell cultures, the final DMSO concentration was 0.25–0.5%.

### Pharmacological growth inhibition assays

Cultured melanoma cells were seeded into 48-well plates. Twenty-four hours after seeding, serial dilutions of individual drugs, drug combinations and/or vehicle (DMSO), as indicated in the text, were added to achieve the desired final concentrations. The doses were 0.004, 0.016, 0.063, 0.25, 1, 4 μM for AZD8055; 0.004, 0.016, 0.063, 0.25, 1, 4, 16 μM for PLX4720 and Dasatinib; 0.063, 0.25, 1, 4, 8, 16 μM for Erlotinib and Gefitinib; 0.25, 0.5, 1, 2, 4, 8, 16 μM for GDC0941, Crenolanib and Lapatinib; 0.5, 1, 2, 4, 8, 16 μM for Canertinib; 0.0016, 0.008, 0.04, 0.02, 1, 5 μM for BEZ235. For growth inhibition, cells were incubated for 72 h and cell viability determined using alamarBlue assay (Bio-Rad, Hercules, CA, USA) as previously described [19]. For Western blot, cells were processed after 24 hr incubation.

### Growth factor pool stimulation

After an overnight starvation, melanoma cells were treated for 10 min with serum-free medium containing or not a pool of human growth factors consisting of 100 ng/ml EGF (Cell Signaling, Danvers, MA, USA), 500 ng/ml recombinant (r) Gas6, 100 ng/ml rNRG1-β1/HRG1-β1 (R&D Systems, Minneapolis, MN, USA) and 100 ng/ml PDGF (Sigma-Aldrich, St.Louis, MO, USA).

### Western blotting

Untreated or treated melanoma cells were processed as described [19] using the primary antibodies listed in Supplementary Table 7.

### Immunohistochemistry

Formalin-fixed paraffin-embedded tissues of melanoma samples were sectioned at 1 μm slide. Immunohistochemical detection was performed as described [19, 58] using antibodies listed in Supplementary Table 7. Slides were scored for staining by a dermatopathologist blinded to clinical outcome and were imaged with a digital camera (AxioCamMrC5, Zeiss, Oberkochen, Germany) using an Axiovert 100 microscope (Zeiss) and a 20x objective.

### Statistical analysis and drug synergy testing

Differences in expression level of RTKs and differences in IC50 values of PLX4720 among subtypes were assessed by ANOVA *p*-values adjusted for multiple testing using the Benjamini-Hochberg false discovery rate. Tukey's post-hoc analysis was performed only when the overall adjusted *p*-values was < 0.05. Association between categorical variables was carried out using Fisher's exact test. All statistical tests were two-sided. Correlation between variables was calculated using Pearson's correlation coefficient. Analyses were conducted in R environment version 3.0.2. For pharmacological growth inhibition assays: generation of fitted lines using the four-parameter nonlinear regression with a sigmoidal dose-response (variable slope) and calculation of IC50 values of dose-response curves were done using Prism v 5.0 software (GraphPad Software, La Jolla, CA, USA); the combination index (CI) between pharmacological inhibitors was established by the Chou and Talalay method [59] using CompuSyn software (Biosoft, Cambridge, UK). CI < 1 was considered synergism.

## SUPPLEMENTARY FIGURES AND TABLES








