# Supplementary material for: A melanoma subtype with intrinsic resistance to BRAF inhibition identified by receptor tyrosine kinases gene-driven classification
Source: Oncotarget. 2015 Jan 30;6(7):5118–33. doi: 10.18632/oncotarget.3007 (PMC4467137; doi:10.18632/oncotarget.3007)
Supplement: Supplementary file 1 [file oncotarget-06-5118-s001.pdf]

SUPPLEMENTARY FIGURES AND TABLES

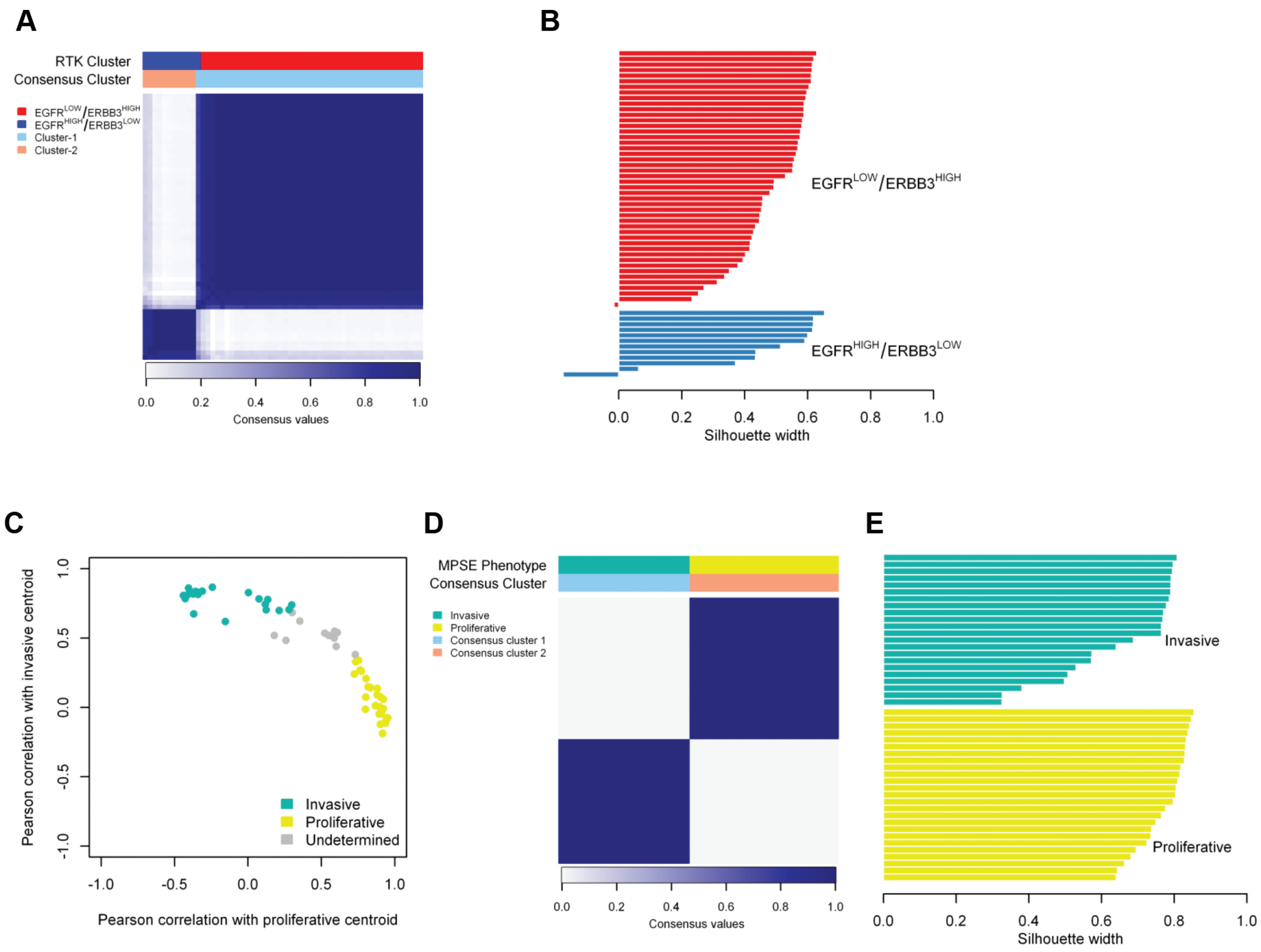

Supplementary Figure S1: Subtype robustness in melanoma cell lines present in the CCLE dataset. **(A)** Consensus clustering using RTK gene expression with the number of clusters fixed at two. The labels assigned by consensus clustering (consensus cluster 1 and 2) are compared to those obtained from hierarchical clustering ($EGFR^{LOW}/ERBB3^{HIGH}$) showing high concordance. Only one sample had a different group assignment**.** **(B)** Silhouette plot analysis of clusters identified by RTK gene expression. Silhouette width is defined as the ratio of each sample's average distance to samples in the same cluster to the smallest distance to samples not in the same cluster. Samples with a positive silhouette best represent each subtype. **(C)** Correlation of each sample with the proliferative or invasive centroids, according to MPSE signature. **(D)** Consensus clustering using the MPSE signature. Labels assigned by consensus clustering and invasive/proliferative phenotype prediction are compared showing perfect concordance. **(E)** Silhouette plot analysis of clusters identified by MPSE.

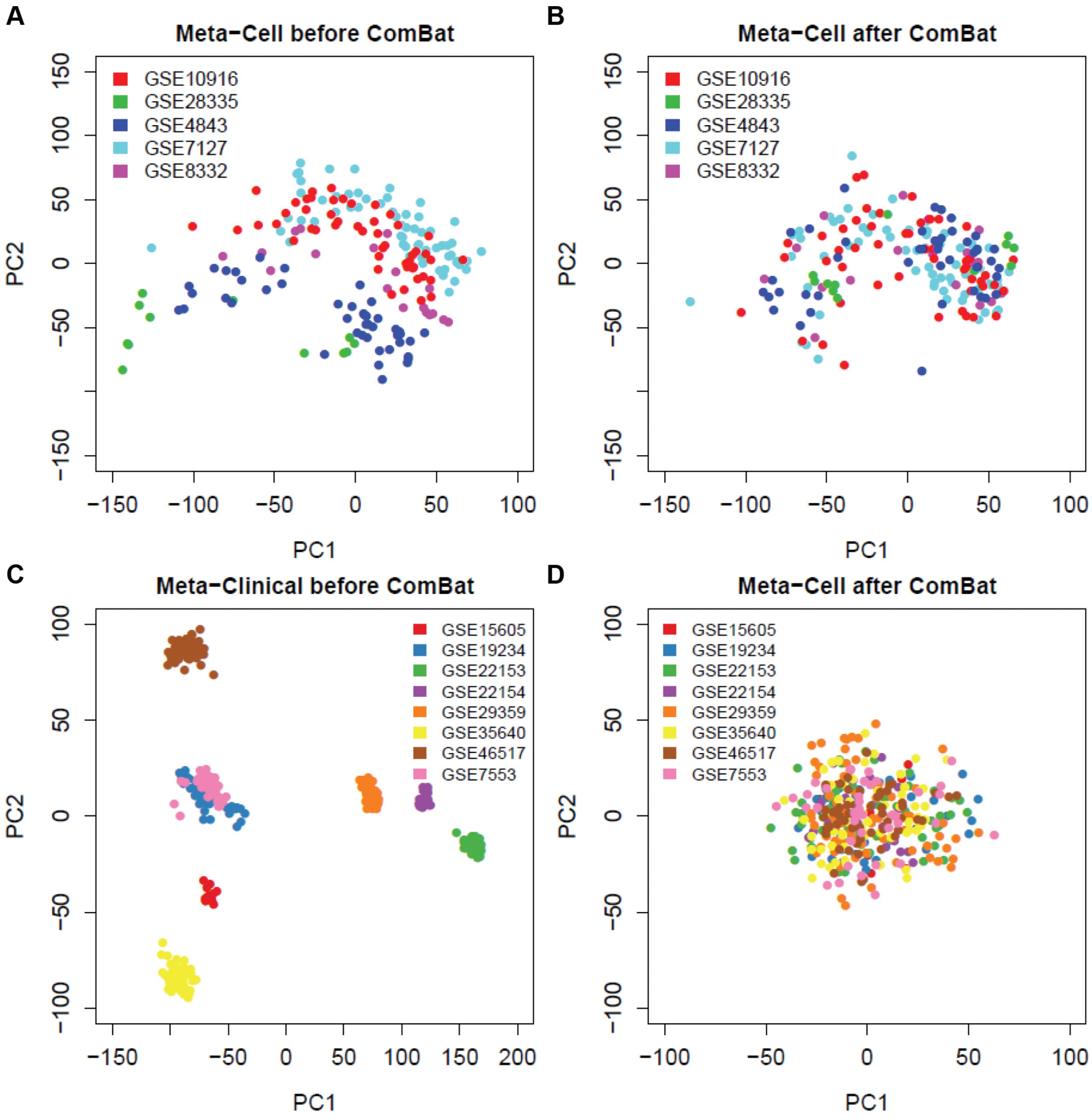

Supplementary Figure S2: Batch effect removal in Meta-cell and Meta-clinical combined expression microarray datasets. **(A)** and **(C)** Principal component analysis of Meta-Cell (A) and Meta-Clinical (C) gene expression data prior to ComBat correction. **(B)** and **(D)** After ComBat correction the technical variations were eliminated in both Meta-Cell (B) and Meta-Clinical datasets (D). Datasets in Meta-Cell were merged at the probe set level, while Meta-Clinical dataset was generated at the gene level due to merging of different microarray platforms.

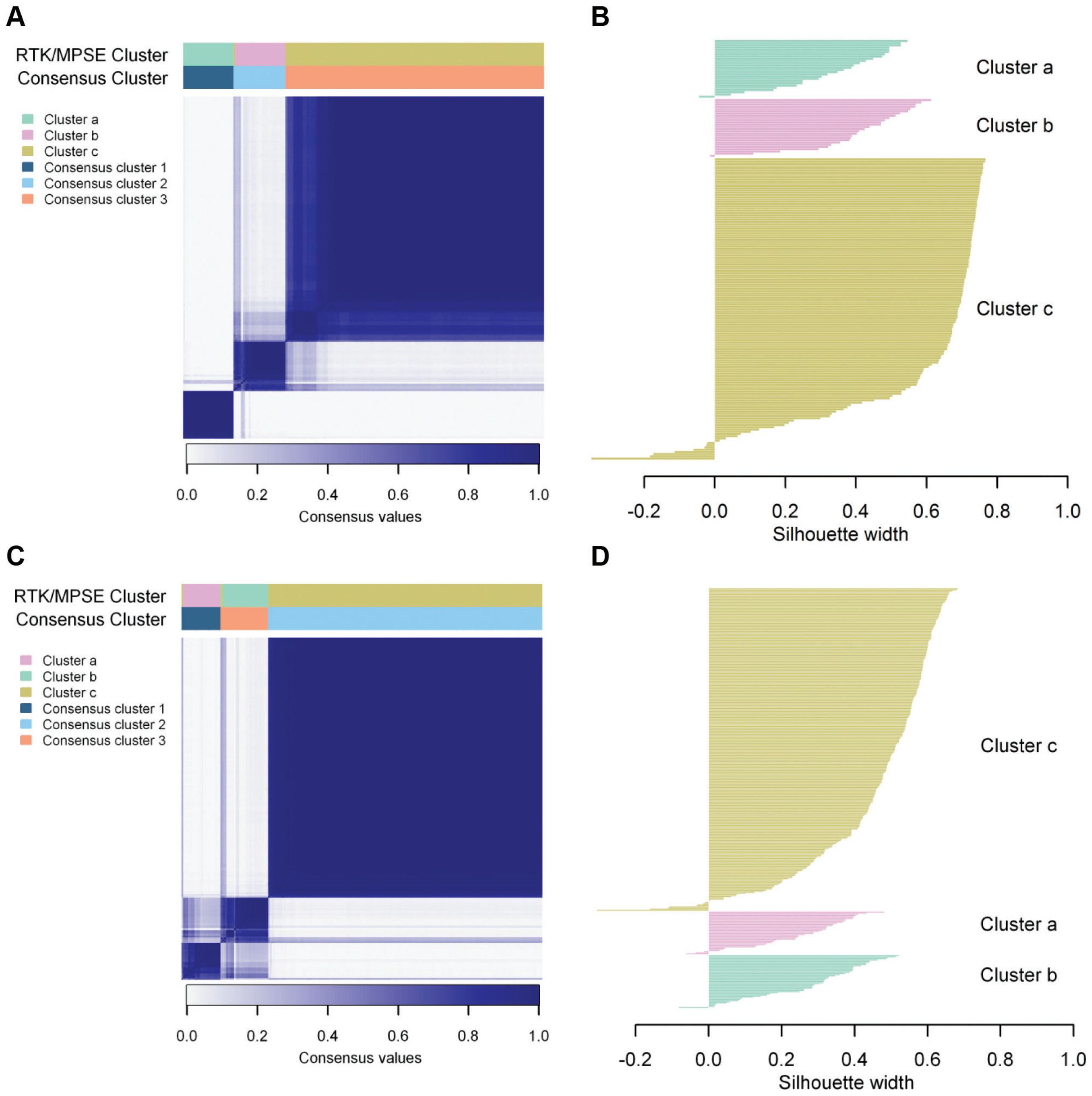

Supplementary Figure S3: Subtype robustness in Meta-cell and Meta-clinical datasets. (A) and **(C)** Consensus hierarchical clustering of Meta-Cell (A) and Meta-Clinical (C) datasets using the RTK/MPSE signature; labels assigned by consensus clustering and hierarchical clustering are compared showing high concordance. **(B)** and **(D)** Silhouette plot of clusters identified by RTK/MPSE signature in the Meta-Cell **(**B) and Meta-Clinical (D) datasets.

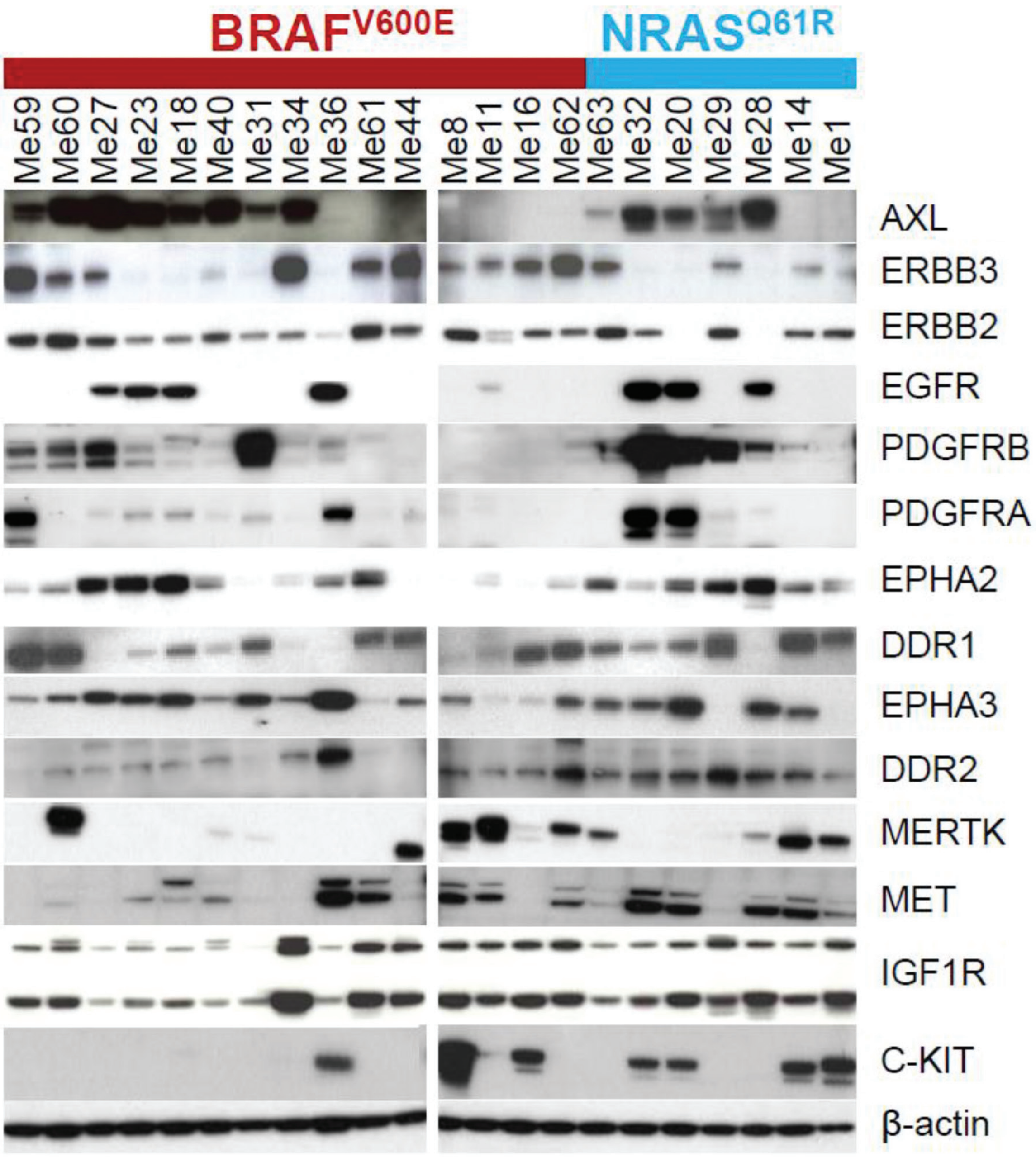

Supplementary Figure S4: Heterogeneity of RTK expression in melanoma cell lines. Western blot analyses of 14 RTKs for the panel of 22 melanoma cell lines. The *BRAF* and *NRAS* mutational status is reported. Expression of AXL, ERBB3, EGFR, PDGFRB, PDGFRA, EPHA2, MERTK is also shown in Figure 3B.

Supplementary Table S1: Samples used in CCLE, Meta-Cell and Meta-Clinical datasets

Supplementary Table S2: Differential expression of genes identified by ClaNC among RTK/MPSE subtypes

Supplementary Table S3: RTK gene expression differences among RTK/MPSE subtypes

Symbol	False Discovery Rate (ANOVA)		
	CCLE	Meta-Cell	Meta-Clinical
**AXL**	1.25E-09	1.37E-25	4.66E-18
DDR2	1.39E-02	1.19E-04	3.11E-01
**EGFR**	1.22E-11	2.99E-36	1.18E-04
**EPHA2**	4.75E-06	1.26E-13	5.18E-02
EPHA3	4.75E-06	1.19E-04	6.23E-07
EPHA4	3.47E-01	6.67E-02	1.42E-01
EPHA5	2.52E-01	5.69E-03	1.04E-09
EPHA7	9.13E-01	3.16E-03	4.43E-01
EPHB1	8.47E-05	1.83E-06	5.51E-07
ERBB2	1.39E-02	2.59E-04	4.74E-01
**ERBB3**	2.76E-16	1.84E-31	6.81E-34
FGFR1	1.02E-04	1.07E-09	7.59E-17
FGFR2	3.18E-02	2.76E-05	1.21E-01
FGFR3	1.10E-01	4.26E-05	1.41E-01
FGFR4	3.42E-03	5.47E-07	4.41E-01
FGFRL1	1.06E-03	8.94E-14	NA
FLT1	1.84E-02	2.93E-12	6.60E-03
IGF1R	8.35E-04	3.69E-03	1.01E-06
IGF2R	3.80E-03	9.33E-03	1.50E-01
INSR	4.17E-02	7.30E-01	9.15E-01
KDR	1.65E-02	7.07E-02	4.37E-03
KIT	3.18E-02	1.68E-03	5.39E-12
**MERTK**	4.93E-05	1.86E-10	2.42E-02
MET	1.78E-02	5.25E-01	4.30E-06
NTRK2	5.47E-02	1.01E-01	1.94E-03
**PDGFRA**	4.07E-08	1.44E-20	5.07E-12
**PDGFRB**	1.87E-07	1.05E-26	1.13E-12
PDGFRL	5.13E-02	8.53E-01	1.76E-03
PTK7	8.01E-01	2.87E-03	3.27E-01
ROR1	1.23E-02	6.18E-03	3.37E-02
RYK	9.82E-01	7.10E-02	2.71E-01
STYK1	2.26E-02	4.76E-13	2.49E-02
TEK	1.12E-04	6.18E-13	3.17E-04
TYRO3	4.32E-05	2.93E-12	4.18E-19

Supplementary Table S4: Features of melanoma cell lines in the panel[1]

Cell line Number[1]	Type	Origin	BRAF	NRAS	p53	PTEN	Subtype
Me18	Metastasis	LN	V600E	wt	wt	Q298Stop[2]	EGFR^{HIGH}/ERBB3^{LOW}_Invasive
Me23	Metastasis	LN	V600E	wt	wt	wt	EGFR^{HIGH}/ERBB3^{LOW}_Invasive
Me27	Metastasis	LN	V600E	wt	C135W[2]	wt	EGFR^{HIGH}/ERBB3^{LOW}_Invasive
Me36	Primary		V600E	wt	wt	Del Exon 6,7,8, P89S[2]	EGFR^{HIGH}/ERBB3^{LOW}_Invasive
Me20	Primary		wt	Q61R	HD[3]	wt	EGFR^{HIGH}/ERBB3^{LOW}_Invasive
Me28	Metastasis	SC	wt	Q61R	HD[3]	wt	EGFR^{HIGH}/ERBB3^{LOW}_Invasive
Me32	Metastasis	LN	wt	Q61R	Y126H[2]	wt	EGFR^{HIGH}/ERBB3^{LOW}_Invasive
Me31	Metastasis	Lung	V600E	wt	wt	HD[c]	EGFR^{LOW}/ERBB3^{HIGH}_Invasive
Me34	Metastasis	LN	V600E	wt	wt	wt	EGFR^{LOW}/ERBB3^{HIGH}_Invasive
Me40	Metastasis	LN	V600E	wt	S127F	P38S	EGFR^{LOW}/ERBB3^{HIGH}_Invasive
Me59	Metastasis	LN	V600E	wt	wt	wt	EGFR^{LOW}/ERBB3^{HIGH}_Invasive
Me60	Metastasis	SC	V600E	wt	wt	del403–409 (Exon 5)[2]	EGFR^{LOW}/ERBB3^{HIGH}_Invasive
Me29	Metastasis	Fibroadipous	wt	Q61R	R213R	wt	EGFR^{LOW}/ERBB3^{HIGH}_Invasive
Me11	Metastasis	LN	V600E	wt	wt	wt	EGFR^{LOW}/ERBB3^{HIGH}_Proliferative
Me16	Metastasis	LN	V600E	wt	wt	wt	EGFR^{LOW}/ERBB3^{HIGH}_Proliferative
Me44	Metastasis	LN	V600E	wt	P94L[2]	R80stop[2]	EGFR^{LOW}/ERBB3^{HIGH}_Proliferative
Me61	Metastasis	LN	V600E	wt	wt	wt	EGFR^{LOW}/ERBB3^{HIGH}_Proliferative
Me62	Metastasis	LN	V600E	wt	wt	wt	EGFR^{LOW}/ERBB3^{HIGH}_Proliferative
Me8	Metastasis	LN	V600E	wt	wt	wt	EGFR^{LOW}/ERBB3^{HIGH}_Proliferative
Me1	Metastasis	LN	wt	Q61R	wt	wt	EGFR^{LOW}/ERBB3^{HIGH}_Proliferative
Me14	Metastasis	LN	wt	Q61R	wt	wt	EGFR^{LOW}/ERBB3^{HIGH}_Proliferative
Me63	Metastasis	LN	wt	Q61R	wt	wt	EGFR^{LOW}/ERBB3^{HIGH}_Proliferative

[1]All primary and metastatic melanoma cell lines were established *in vitro* from surgical specimens of primary lesions ($n = 2$) or lymph node, subcutaneous or visceral metastases ($n = 20$) removed from patients admitted for surgery to our Institution and not previously treated. Cell lines are numbered according to the list reported in Sensi M. et al. J. Invest. Dermatol. 2011; 131:2448–57. Me59–63 were not included in the previous list. All lesions were histologically confirmed to be cutaneous malignant melanomas.

[2]Homozygous

[3]Homozygous Deletion

Supplementary Table S5: Effect of all drugs according to RTK/MPSE subtype on CCLE melanoma cell lines

Compound	Targets	Company	False Discover Rate (ANOVA)	
			All cell lines[1]	BRAF-V600E/D[1]
17-AAG	HSP90	Bristol-Myers Squibb	0.798	0.700
AEW541	IGF-1R	Novartis	0.851	0.700
AZD0530	Src, Abl/Bcr-Abl, EGFR	AstraZeneca	0.047	0.078
AZD6244	MEK	AstraZeneca	0.023	0.030
Erlotinib	EGFR	Genentech	0.755	0.700
Irinotecan	Topoisomerase I	Pfizer	0.755	0.720
L-685458	Gamma Secretase	Merck Sharp & Dohme	0.291	0.463
Lapatinib	EGFR, HER2	GlaxoSmithKline	0.755	0.700
LBW242	IAP	Novartis	0.210	0.690
Nilotinib	Abl/Bcr-Abl	Novartis	0.500	0.700
Nutlin-3	MDM2	Roche	0.798	0.720
Paclitaxel	beta-tubulin	Bristol-Myers Squibb	0.488	0.700
Panobinostat	HDAC	Novartis	0.488	0.308
PD-0325901	MEK	Pfizer	0.328	0.030
PD-0332991	CDK4/6	Pfizer	0.133	0.700
PF2341066	c-MET, ALK	Pfizer	0.133	0.700
PHA-665752	c-MET	Pfizer	0.798	0.700
PLX4720	RAF	Plexxikon	0.076	0.001
RAF265	Raf kinase B, KDR	Novartis	0.755	0.391
Sorafenib	Flt3, C-KIT, PDGFRbeta, RET, Raf kinase B, Raf kinase C, VEGFR-1, KDR, FLT4	Bayer	0.667	0.700
TAE684	ALK	Novartis	0.023	0.307
TKI258	EGFR, FGFR1, PDGFRbeta, VEGFR-1, KDR	Novartis	0.798	0.720
Topotecan	Topoisomerase I	GlaxoSmithKline	0.798	0.700
ZD-6474	EGFR	AstraZeneca	0.798	0.873

[1]For each drug the number of cells tested is different. Considering all samples, the number of cell lines used in the analyses goes from a minimum of 21 to a maximum of 28. Considering only samples with a BRAFV600 mutation , the number of cells lines goes froma a minimum of 13 to a maximum of 19.

Supplementary Table S6: Information of patients and of melanoma specimens used in immuno-histochemical analysis

Supplementary Table S7: Antibodies used in the study

Antigen protein	Type	Company	Application[1]
AKT	polyclonal (C-terminus) rabbit	Cell Signaling (9272)	IB
AKT-S473	monoclonal rabbit	Cell Signaling (4060)	IB
AXL	polyclonal (C-terminus) goat	Santa Cruz Biotechnology (sc-1096)	IB
AXL	polyclonal (extracellular domain) goat	R&D Systems (AF154)	IHC
AXL-Y702	monoclonal rabbit	Cell Signaling (5724)	IB
c-KIT	polyclonal(C-terminus) rabbit	Dako (A4502)	IB
DDR1	monoclonal rabbit	Cell Signaling (5583)	IB
DDR2	polyclonal (N-terminus)	Santa Cruz Biotechnology (sc-7555)	IB
EGFR	monoclonal (cytoplasmatic domain) rabbit	Cell Signaling (4267)	IB, IHC
EGFR-Y1068	monoclonal rabbit	Cell Signaling (3777)	IB
EPHA2	polyclonal (C-terminus) rabbit	Santa Cruz Biotechnology (sc-924)	IB
EPHA2-Y588	monoclonal rabbit	Cell Signaling (12677)	IB
EPHA3	polyclonal (C-terminus) rabbit	Santa Cruz Biotechnology (sc-919)	IB
ERBB2	monoclonal rabbit	Cell Signaling (4290)	IB
ERBB3	monoclonal (C-terminus) mouse	Santa Cruz Biotechnology (sc-7390)	IB
ERBB3-Y1289	monoclonal rabbit	Cell Signaling (4791)	IB
ERK	polyclonal rabbit	Promega (V1141)	IB
ERK-T183/Y185	monoclonal mouse	Sigma-Aldrich (M8159)	IB
FAK	polyclonal (N-terminus) rabbit	Santa Cruz Biotechnology (sc-557)	IB
FAK-Y925	polyclonal rabbit	Cell Signaling (3284)	IB
FGFR1	monoclonal (C-terminus) rabbit	Cell Signaling (9740)	IB
IGF1R	monoclonal (C-terminus) rabbit	Cell Signaling (9750)	IB
MERTK	monoclonal (extracellular domain) mouse	Cell Signaling (9178)	IB
MET	monoclonal mouse	Cell Signaling (3127)	IB
MITF	monoclonal mouse	Dako (M3621)	IB, IHC
Paxillin-Y118	polyclonal rabbit	Invitrogen (44–722)	IB
PDGFRA	monoclonal (extracellular domain) rabbit	Cell Signaling (5241)	IB
PDGFRB	polyclonal rabbit	Santa Cruz Biotechnology (sc-432)	IB
PDGFRB-Y571	monoclonal rabbit	Cell Signaling (4549)	IB
S6	monoclonal mouse	Cell Signaling (2317)	IB
S100	polyclonal rabbit	Dako (Z0311)	IHC
S6-S235/236	monoclonal rabbit	Cell Signaling (4858)	IB
S6-S240/244	monoclonal rabbit	Cell Signaling (5364)	IB
SRC-Y416	polyclonal rabbit	Cell Signaling (2101)	IB
β-actin	polyclonal (C-terminus) rabbit	Sigma-Aldrich (A2066)	IB

[1]IB, immunoblotting; IHC, immunohistochemistry
